# Prognostic Value of Elevated Pre-treatment Serum CA-125 in Epithelial Ovarian Cancer: A Meta-Analysis

**DOI:** 10.3389/fonc.2022.868061

**Published:** 2022-04-07

**Authors:** Qingyi Wang, Xiaoling Feng, Xiaofang Liu, Siyu Zhu

**Affiliations:** ^1^ Department of First Clinical Medical College, Heilongjiang University of Chinese Medicine, Harbin, China; ^2^ Department of Gynecology, The First Affiliated Hospital of Heilongjiang University of Chinese Medicine, Harbin, China

**Keywords:** CA-125, ovarian cancer, prognosis, meta-analysis, systematic review, neoadjuvant chemotherapy, overall survival, progress-free survival

## Abstract

**Background:**

CA-125 is a clinical biomarker with predictive effect on the prognosis of different cancers. Numerous clinical trials have been conducted to investigate the possibility of using the pretreatment level of CA-125 to predict the prognosis of epithelial ovarian cancer (EOC). However, its value in predicting prognosis remains controversial. The purpose of this meta-analysis was to assess the predictive value of pretreatment CA-125 levels for prognosis in EOC patients.

**Methods:**

We searched the EMBASE, Cochrane library, PubMed and Web of Science databases for studies published up to 3 December 2021, according to specific inclusion and exclusion criteria. The clinical studies that were included investigated the relationship between pretreatment CA-125 levels and ovarian cancer prognosis. Combined hazard ratios (HR) of overall survival (OS) and progression-free survival (PFS) reported in the studies were compared and analyzed using fixed-effects/random-effects models. Sensitivity analysis was used to assess study stability, while Egger’s and Begg’s tests were used to assess publication bias.

**Results:**

This meta-analysis included 23 studies published in 2004 - 2021 with a total of 10,594 EOC patients. Comprehensive analysis demonstrated that the serum level of CA-125 before treatment was significantly correlated with overall survival (OS: HR=1.62, 95%CI=1.270-2.060, p<0.001) and progression-free survival (PFS: HR=1.59, PFS: HR=1.59, 95%CI=1.44~1.76, p<0.001). After comparing data from different FIGO stages and treatments, we discovered that a high pre-treatment serum CA-125 level was associated with a low survival rate.

**Conclusion:**

According to the results of this study, a higher pre-treatment serum CA-125 level is associated with poor survival outcomes, which can be utilized to predict the prognosis of EOC patients. Pre-treatment serum CA-125 level might provide reliable basis for predicting the risk of EOC disease progression. This study is registered with the International Prospective Register of Systematic Reviews (CRD42022300545).

**Systematic Review Registration:**

https://www.crd.york.ac.uk/prospero/display_record.php?RecordID=300545, identifier [CRD42022300545].

## Introduction

Ovarian cancer (OC) is one of the most common malignant tumors in the world, with the highest fatality rate among gynecological malignant tumors with a high degree of malignancy. The 5-year survival rate is less than 45% (1, 2). There are an estimated 290,000 newly diagnosed cases and 180,000 deaths every year around the world (3). Only less than 40% of the women with OC are diagnosed at an early stage due to the lack of typical clinical manifestations and reliable screening approaches. Nowadays, taxane and platinum-based chemotherapy after aggressive cytoreduction therapy is the accepted standard of treatment (4, 5). Despite receiving standard treatment, 75-80% of patients with advanced OC relapsed within 5 years of the initial diagnosis. Various markers have been used in early diagnosis or prognosis prediction, including D-dimer (6), Human epididymis protein 4 (HE4) (7), MMP-2, Survivin, Ki-67, etc. (8, 9). However, the prognosis of OC patients remains poor. Therefore, discovering an accurate and cost-effective predictive approach to evaluate the prognosis of EOC patients before treatment is importance when making the treatment plans.

CA-125 is a transmembrane macromolecule glycoprotein secreted by coelomic epithelial cells. It has become a consensus that CA-125 plays an important role in the prognosis of OC (10–12), and the role of CA-125 in tumorigenesis, metastasis and CA-125-targeted therapy has received great attention. CA-125 has high sensitivity and low specificity because inflammatory environments may promote CA-125 secretion, and non-tumor cells, such as mesothelial cells, and also secrete CA-125 in pro-inflammatory environments (such as ascites) (13). In ovarian cancer, preoperative CA-125 level varies greatly among individuals and may be significantly increased in some patients, which is beneficial for early identification and diagnosis of the tumor. At the same time, CA-125 levels remain in the normal range in more than half of the patients before surgery, making diagnosis and decision-making challenging and confusing for these patients. Numerous studies have been conducted in recent years to investigate the cut-off value of serum CA-125 before treatment and its role as a non-invasive factor in assessing the correlation with EOC prognosis (14–23). Elevated CA-125 level is considered to be a high risk factor for recurrence and death (24–27), however the results were inconsistent. Therefore, we performed this meta-analysis to determine the prognostic significance of the CA-125 level before treatment in EOC patients.

## Materials and Methods

### Search Strategy

This meta-analysis was conducted in accordance with the preferred reporting items for systematic reviews and meta-analyses (PRISMA) (28). We thoroughly searched the PubMed, Web of Science, Embase and Cochrane Library databases for eligible studies published as of December 3, 2021. The following search phrases were used for literature retrieval: (ovarian cancers OR ovarian neoplasms OR OC OR ovarian tumor OR cancer of ovary OR ovary cancer) AND (CA-125 OR CA 125 OR CA-125 OR Carbohydrate antigen 125 OR Cancer antigen 125) AND (prognosis OR prediction). Detailed search strategies are shown in the online supplementary material. We also searched references of the included articles and grey literature to identify if there were any potential articles to be included. Since the data used in this article were extracted from previous literature, no patient consent or ethical approval was required.

### Inclusion and Exclusion Criteria

Inclusion and exclusion criteria: The inclusion criteria were as follows: (1) EOC was diagnosed according to pathological examination or current clinical guidelines; (2) serum CA-125 level was collected before chemotherapy, surgery and neoadjuvant chemotherapy with an explicit cut-off value; (3) articles investigating the relationship between CA-125 and EOC prognosis [including hazard ratio (HR) and corresponding 95% confidence interval (CI) for overall survival (OS) and/or disease-free progression (PFS)], or providing sufficient data to calculate these indicators, including the clinical pathological parameters; (4) published as full-text papers; and (5) the articles were written in English. The exclusion criteria were as follows: (1) reviews, conference abstracts, letters, editorials, expert opinions, case reports, abstracts, and reviews; (2) basic research or animal studies; (3) duplicate publications; and (4) studies on the tumors originating in the gastrointestinal tract and having metastasis to the ovary.

### Data Collection, Extraction and Quality Assessment

Two investigators independently collected corresponding data from eligible studies, and any disagreements were resolved through discussions with a third investigator. The following information was extracted: (1) study characteristics including the first author, country of origin, year of publication, number of patients, duration of follow-up, and method of survival analysis; (2) patient characteristics including geographical area, age, metastasis, FIGO stage, treatment and cut-off value; and (3) survival measures including HRs of OS, PFS, DFS and their 95% CIs. The HRs were extracted from multivariate or univariate analyses. If HR values were not provided in the original text, Kaplan-Meier survival curves were used to determine HR values and corresponding CIs (Engauge Digitizer version 10.8). In this meta-analysis, the primary and secondary outcomes were OS and PFS. E-mails were also sent to the corresponding author for the requested data.

Newcastle-Ottawa Scale (NOS) (29) was used to assess the methodological quality of included studies. The studies were graded from 0 to 9 based on cohort selection, comparability, and results. Studies having a score of 6 or above were considered to be of high-quality research. Any disagreements were resolved by a senior researcher.

### Statistical Analysis

Pooled HRs and 95% CIs were calculated to assess the relationship between pretreatment CA-125 level and prognosis. The heterogeneity of the included studies was assessed using Cochran’s q-test and Higgins I^2^ statistic. Data were combined using a random-effects model (DerSimonia-Laird method) if I^2^>50% or p<0.10 (indicating significant heterogeneity among studies); otherwise a fixed-effects model (Mantel-Haenszel method) was used. To identify sources of heterogeneity between studies, subgroup analyses were conducted based on geographic region, treatment method, and FIGO stage. Furthermore, sensitivity analysis was conducted to assess the impact of individual study data on HRs for OS and PFS. All statistical calculations were performed using STATA MP16 (Stata, College Station, TX, USA). In all the analyses, p<0.05 (two-sided) indicated a statistically significant difference.

## Results

### Search Results and Characteristics of the Studies

**Figure 1:**
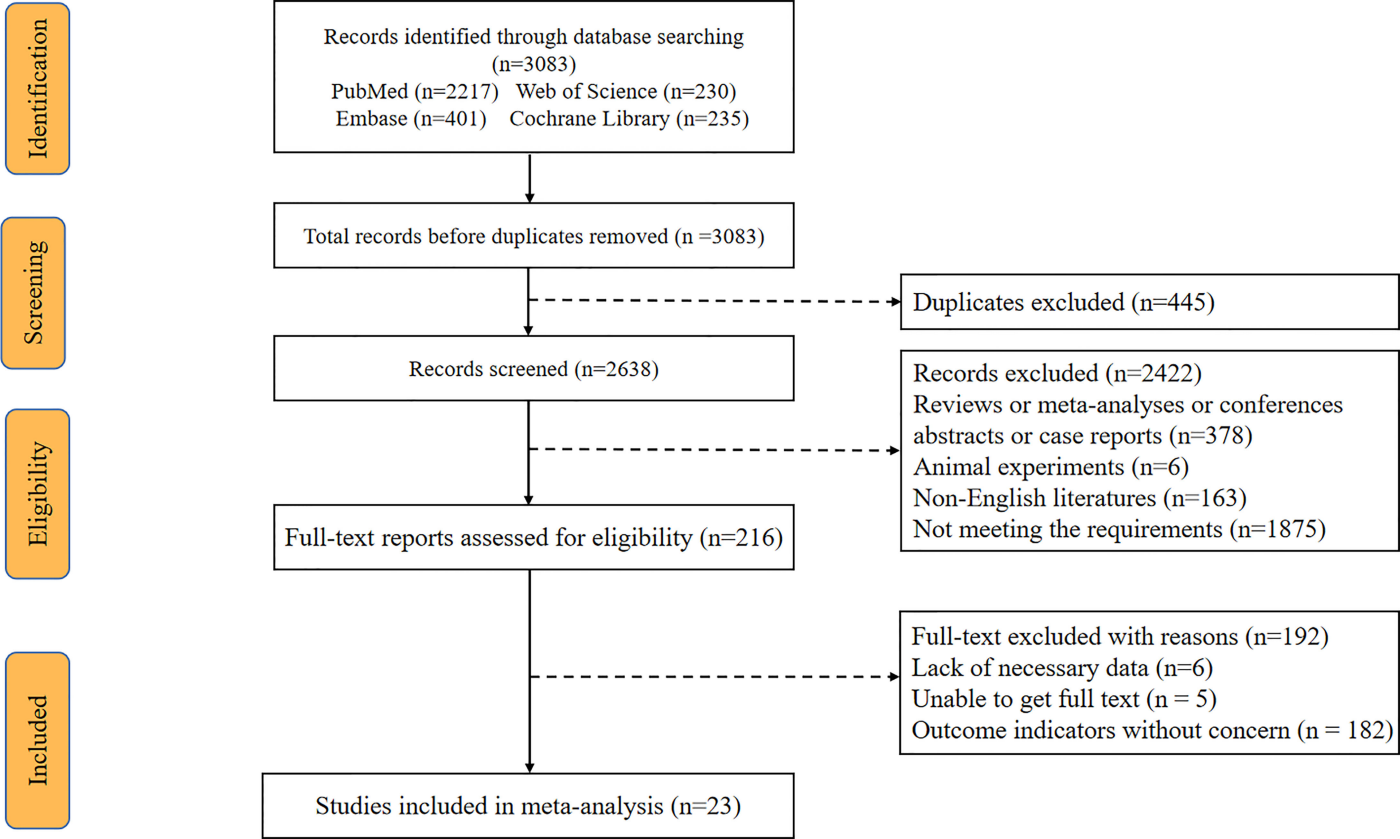
Flow diagram of the study selection process.

A total of 3083 articles were retrieved from the system’s database, with 445 of them being eliminated owing to dataset duplication. Another 2422 articles were eliminated after analyzing their titles and abstracts. Afterward, 216 articles were reviewed using full-text reading, with 192 of them being eliminated for the following reasons: Six studies lacked required data; five studies were not accessible in full text, and 181 studies did not provide the desired result. There are a total of 24 eligible articles, one which was published in 1995 and was excluded because it was too old (20). Finally, our meta-analysis includes 23 articles published between 2004 and 2021. The flow chart of literature search is shown in **Figure 1**. The 23 eligible studies included a total of 10,594 patients, with numbers of patients ranging from 30 to 3,474. There were 17 studies exploring the relationship between CA-125 level and OS, and 13 studies providing the relationship between CA-125 and PFS. The cutoff value was between 33 and 1200. There were 20 retrospective cohort studies and 3 prospective cohort studies out of the 23 eligible studies. Four studies were from Europe, 10 from Asia, 2 from the Americas and 1 from Oceania. In 13 studies, HR was recorded in univariate analysis. In 10 trials, HR was measured using multivariate analysis, while in 5 studies, HR was obtained using survival curves. The main characteristics of the 23 included studies are shown in **Table 1**. The NOS scores of all the studies were between 6 and 9. The included studies all had a high NOS score ≥ 6.

**Table 1:** Major features of included studies in this meta-analysis.

Author	Year	Country	Study design	Sample size	Age, years Median[range]	Cut-off value (U/mL)	FIGO stage	Treatment	Survival analysis	NOS score
BACHMANN1	2021	Germany	Retrospective	136	62 (38-81)	500	III-IV	NACT+ Surgical resection+Chemotherapy	OS	7
Ay	2021	Turkey	Retrospective	230	55.5 (19–84)	385	I-III	Surgical resection+Chemotherapy	OS	9
Yamada	2020	Japan	Retrospective	199	60.0 (33–90)	200	I-IV	NACT+Surgical resection+Chemotherapy	OS	9
Wang	2020	China	Retrospective	174	59 (17-83)	357.9	I-IV	Surgical resection+Chemotherapy	OS	9
Tang	2020	China	Retrospective	214	50 (20-88)	34	I-IV	NACT+Surgical resection+Chemotherapy	OS;PFS	7
Salminen	2020	Finland	Prospective	122	66 (38-82)	33	I-IV	NACT+Surgical resection	PFS	8
Potenza	2020	Italy	Prospective	426	60 (36-83)	425	I-IV	Surgical resection+Chemotherapy	PFS	8
Lin	2020	China	Retrospective	326	54 (20-89)	35	I-IV	Surgical resection+Chemotherapy	PFS	9
Kim	2020	Korea	Retrospective	107	56.6 ± 10.1	700	I-IV	NACT+Surgical resection	PFS	9
Kang	2020	Korea	Retrospective	84	49.0 ± 15.3	880	I-IV	NACT+Surgical resection+Chemotherapy	OS;PFS	8
Chen	2019	China	Retrospective	108	51 (27–75)	1200	III-IV	NACT+Surgical resection+Chemotherapy	OS;PFS	7
Maria Lee	2016	Korea	Retrospective	233	Adult	500	I-IV	Surgical resection+Chemotherapy	OS;PFS	6
Menczer	2015	Israel	Prospective	114	61.2	35	I-III	Surgical resection+Chemotherapy	OS;PFS	7
Tian	2009	America	Retrospective	3474	57	35	III-IV	Surgical resection+Chemotherapy	OS;PFS	7
A.Prat	2008	Spain	Retrospective	96	59	10	III-IV	Surgical resection+Chemotherapy	OS;PFS	8
Paramasivam	2005	Australia	Retrospective	518	Adult	30	I-III	Surgical resection+Chemotherapy	OS	8
SANTALA	2004	Finland	Retrospective	55	53 (24-73)	170	I-IV	Surgical resection+Chemotherapy	OS	8
NAKAMURA	2016	Japan	Retrospective	30	54.9 (33–78)	722	III-IV	NACT+Surgical resection+Chemotherapy	OS	7
PETRI	2006	Denmark	Retrospective	118	59 (32–78)	65	I-III	Surgical resection+Chemotherapy	OS	8
Ayhan	2021	Turkey	Retrospective	71	58 (23–78)	101	NA	Surgical resection+Chemotherapy	OS	7
CK Lee	2011	Australia	Prospective	955	Adult	100	III IV	Surgical resection+Chemotherapy	PFS	7
Chee K	2011	Australia	Retrospective	886	24-82	100	I-IV	Chemotherapy	PFS	7
Costa	2016	Brazil	Retrospective	209	Adult	100	I-IV	Surgical resection+Chemotherapy	OS	8

### The Effect of CA-125 Level on the Prognosis of OS in EOC Patients

**Figure 2:**
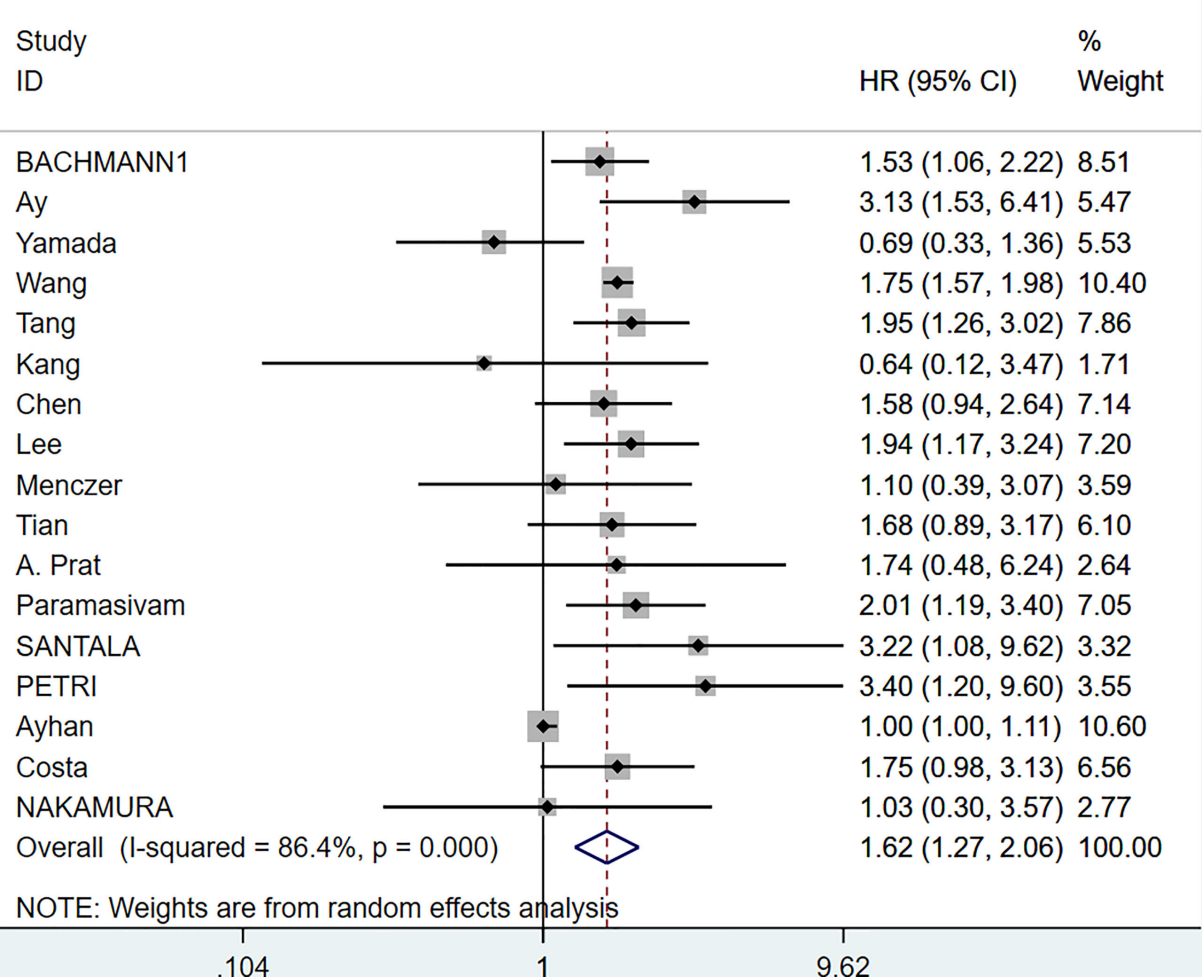
Forest plot of the association of CA-125 with OS in EOC cancer patients. CI, confidence interval; HR, hazard ratio.

**Figure 3:**
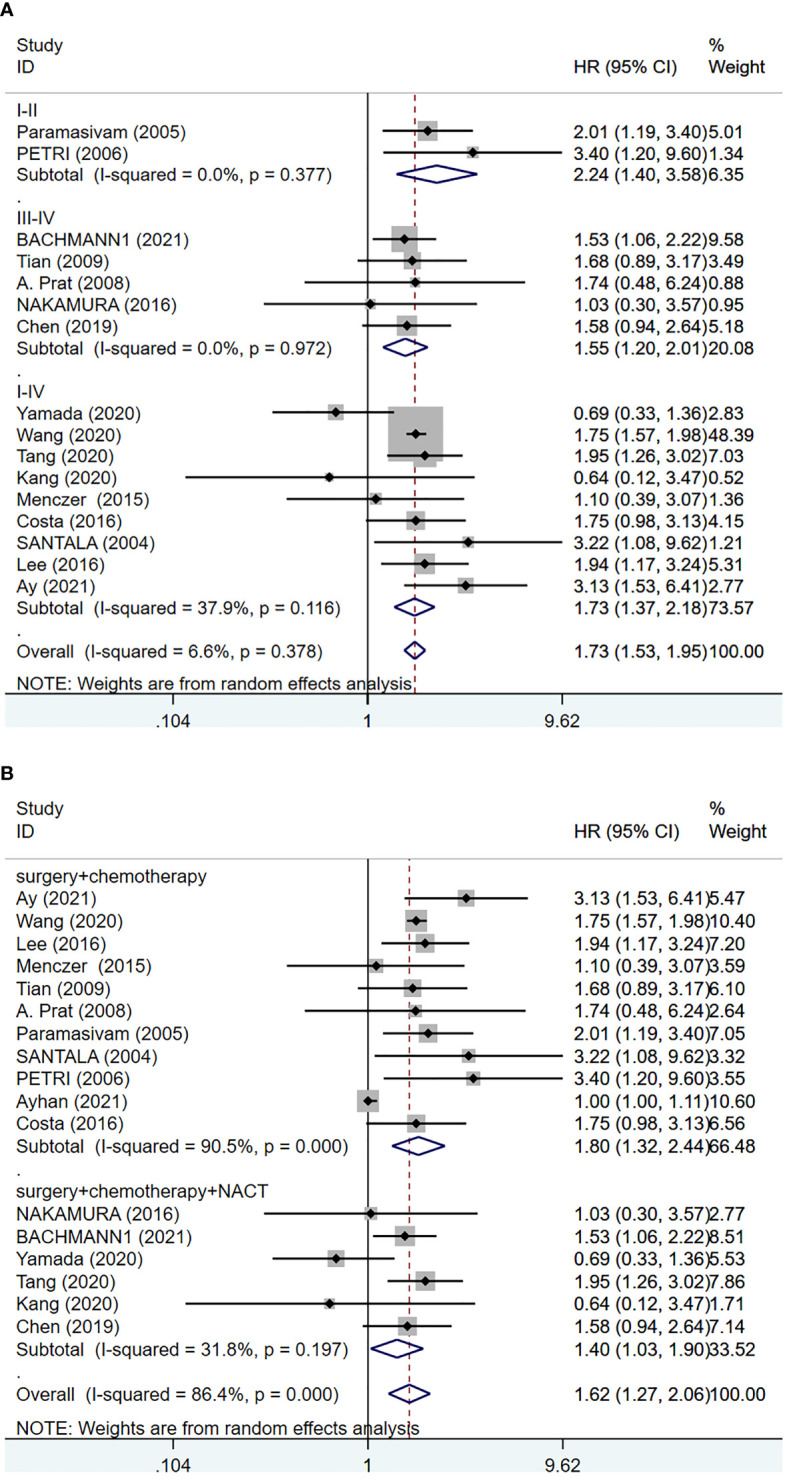
Forest plot of the relationship between CA-125 level and clinical factors in EOC patients. **(A)** FIGO stage (I-II versus III-IV versus I-IV); **(B)** treatment (surgery + chemotherapy versus surgery + chemotherapy + NACT).

We analyzed the data from 6063 EOC cancer patients in 17 studies (6, 14, 15, 17–19, 23, 24, 26, 27, 30–36) to determine the relationship between pretreatment serum CA-125 level and OS. A random-effects model was employed based on the results of the heterogeneity analysis (I^2^ = 86.40%, p <0.001). The results presented that increased serum CA-125 level before treatment was associated with poorer OS (HR=1.62, 95%CI=1.270-2.060, p<0.001, **Figure 2** and **Table S2**), with a statistically significant difference. The heterogeneity of HR was significant in the OS group, thus subgroup analyses were performed based on FIGO stage, and treatment strategy to identify the sources of heterogeneity. Since one of the studies did not explicitly state the FIGO staging (33), no subgroup analysis of FIGO staging was performed. The results suggested that higher heterogeneity might be attributed to differences in geographic regions, tumor stages, and treatment strategies. However, the final results showed that higher pre-treatment CA-125 level was consistently correlated with poorer OS regardless of the grouping (**Figure 3** and **Table S2**).

### The Predictive Effect of CA-125 Level on the Prognosis of PFS in EOC Patients

**Figure 4:**
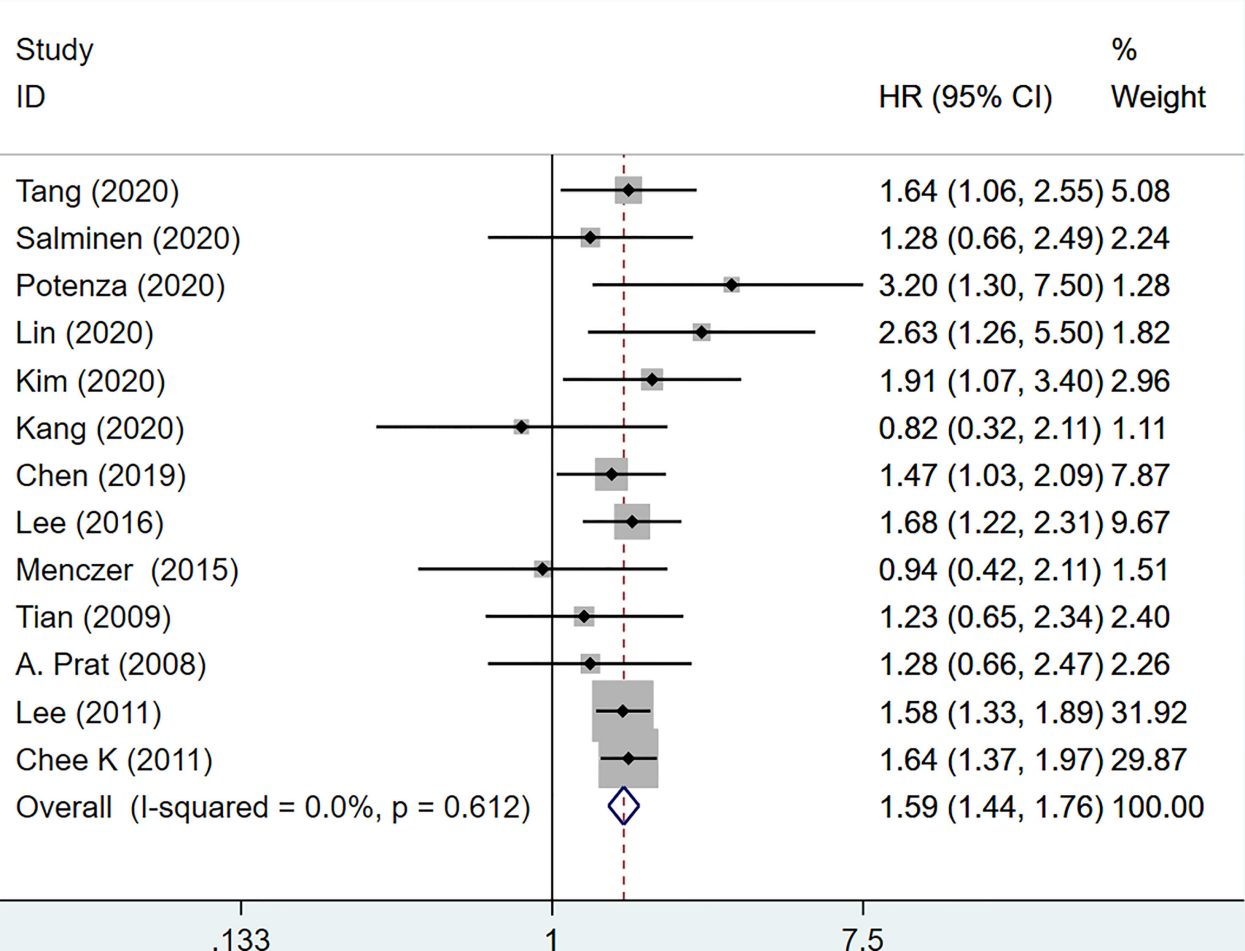
Forest plot of the association of CA-125 level with PFS in EOC patients. CI, confidence interval; HR, hazard ratio.

Data from 13 studies with a total of 7145 patients were retrieved and utilized in the PFS analysis (16–18, 21, 22, 25, 26, 30–32, 36–38). The integrated data showed that increased serum CA-125 level before treatment was associated with poor PFS (HR=1.59, 95%CI=1.44-1.76, p<0.001, **Figure 4**). There was no heterogeneity between studies (I^2^ = 0.0%, p=0.612). Therefore, a fixed-effects model was used. No subgroup analysis was done due to the small number of studies reporting PFS.

### Sensitivity Analysis

Sensitivity analysis was carried out after merging the included literatures. After removing the included literatures one by one, the effect size in the remaining literatures were combined again. The results showed that there was no significant change in the combined HR and 95% CI, indicating that the results of this meta-analysis are relatively stable and reliable (**Figure S1**).

### Publication Bias

Begg’s and Egger’s tests were used to test whether there was publication bias in the included literature. **Figure S2** illustrates that when OS was evaluated for publication bias, Egger’s test revealed a statistically significant difference (p<0.05), indicating that there was publication bias in the results of this study. The reason might be that the authors chose to withdraw the manuscript, delay or omit publication because the outcomes was regarded as negative and meaningless, resulting in fewer reports of relevant results being published. In order to further evaluate the impact of publication bias on the results, trim-and-fill method was used. After one missing study was added, it was found that there was no significant change in the combined effect size, the cut-and-fill method yielded an HR of 1.573 (95%CI: 1.243-1.990). This result indicated that the impact of publication bias was small and the results were relatively stable. The results of other studies showed no statistical significance, suggesting that there was no publication bias. The funnel plot for the trim-and-fill method is shown in **Figure S3**.

## Discussion

Previous studies have proven the relevance of pre-treatment CA-125 levels in predicting EOC prognosis, and this has been consistently verified in guiding clinical practice and prolonging patient survival. However, since there is no consensus on the fixed cut-off value for pre-treatment serum CA-125 concentration, resulting in inconsistent and inconclusive findings, we reviewed published articles to assess the potential for clinical application of pre-treatment CA-125 in EOC. To the best of our knowledge, this is the first systematic review to investigate the prognostic value of pre-treatment CA-125 in EOC patients. In this study, we sought to explore whether the pre-treatment serum CA-125 level could be used as a prognostic indicator of EOC to help predict the best cytoreductive surgery, reduce surgical complications and economic burden, and better evaluate the prognosis of EOC patients. We collected data from 23 studies involving 10,594 patients, and pooled analysis showed that elevated pre-treatment serum CA-125 level was significantly associated with poor prognosis in patients with EOC. In addition, subgroup analysis was performed according to FIGOstage and treatment. The results of all subgroup analyses showed that there was no significant difference in prognosis across different subgroups (p>0.05). The pre-treatment serum CA-125 level had predictive significance for the prognosis regardless of whether the tumor was at an early or advanced stage or if NACT was employed. Elevated CA-125 may be a strong prognostic marker for disease progression, tumor recurrence, and long-term survival outcomes including OS and PFS.

In recent years, the tumor microenvironment has received increasing attention in the field of tumor immunology. The mechanism of CA-125 affecting prognosis may be related to the promotion of metastasis of cancer cells to other sites. CA-125 is a repeated peptide epitope of the mucin MUC16 (39), which binds with high affinity to Mesothelin, a protein lining the peritoneal mesothelium (40), and promotes the attachment of cancer cells to the mesothelial lining, leading to peritoneal metastasis of ovarian cancer cells (41). This mechanism is thought to be related to the interaction of phosphorylation of MMP-7 and p38 mitogen-activated protein kinase (MAPK), increased expression of β-catenin, and translocation of p120ctn (42). In addition, MUC16 has been shown to inhibit the innate immune response to OC cells by directly inhibiting natural killer (NK) cells, helping cancer cells evade the host immune response. The mechanism of action is as follows: MUC16 down-regulates CD16 on NK cells and interacts with Siglec-9 on its surface to inhibit the formation of synapses between NK cells and ovarian cancer cells (41, 43–45), thus inhibiting the anticancer immune response. Increased and decreased serum CA-125 levels are associated with progression and regression of EOC (13). CA-125 at two different concentrations had a significant effect on OC cell migration, and the stimulating effect of CA-125 on cell migration appears to be mediated through the Wnt signaling pathway. Dickkopf-related protein-1 (DKK-1), a Wnt antagonist, has been shown to inhibit cancer cell migration, and its expression in OC tissues is lower than in normal tissues. The majority of OC patients have high serum CA-125 levels, and CA-125 enhances ovarian cancer cell migration by decreasing DKK-1 expression and activating the serum- and glucocorticoid-regulated kinase 3 (SGK3)/forkhead box O3 pathway (FOXO3). CA-125 induces metastasis of OC cells to axillary and inguinal lymphoid tissues (12, 46), resulting in a poor prognosis. CA-125 may play a wide range of rolesin the humoral immunosuppression of cancer by directly binding to a subset of tumor-targeting antibodies and blocking their immune effector functions (47). Hence, elevated CA-125 implies a predominance of tumor-promoting activity in the tumor microenvironment, which ultimately leads to a poor prognosis.

Several previous meta-analyses and retrospective analyses have investigated at the prognostic role of CA-125 in solid tumors. Overexpression of MUC16 is associated with poor prognosis in various malignancies (48–51). CA-125 was found to be an excellent prognostic indicator in a recent investigation on non-gynecologic cancer metastasis to the ovary (52), and another study revealed that CA-125 was a prognostic factor for survival in patients with gynecologic tumor brain metastases (53). Several other studies have also shown that pre-treatment serum CA-125 is an independent prognostic factor in patients with bladder urothelial carcinoma (UCB) (54), pancreatic ductal adenocarcinoma (55), and renal cell carcinoma (56). Here, we demonstrate the prognostic efficacy of CA-125 relative to OS and PFS in EOC, which is consistent with findings in other cancer types.

Due to the biological heterogeneity of EOC, which is not a single disease entity but a group of heterogeneous tumors with different clinicopathological features, different pre-treatment CA-125 levels have been found in patients with different EOC subtypes, such as low levels in mucinous and clear cell carcinomas. Although the included studies did not use a consistent cut-off value for pre-treatment serum CA-125 concentration, most studies found that higher levels of CA-125 in the normal range were associated with a higher risk ratio for recurrence or death (57).

The society of gynecologic oncology and the American society of clinical oncology consider that women with high perioperative risk or a low probability of tumor cytoreduction to a residual lesion <1cm (ideally no lesion visible) should receive NACH. Women who are eligible for PDS and have potentially resectable disease may receive either NACH or PDS. However, PDS is preferred if the likelihood of achieving cytoreduction to <1cm (ideally no visible disease) at an acceptable incidence is high (58). Many studies have been conducted to determine whether pre-treatment serum CA-125 predicts optimal PDS or IDS in patients with EOC, but the results have been inconsistent.

The study by Merlo et al. indicated that higher pre-treatment CA-125 levels were less likely to achieve optimal cytoreduction. At the cut-off value of 500IU/ml, 58% of patients with advanced EOC achieved complete or at least optimal cytoreductive surgery, and at the cut-off value of 250IU/ml, the probability increased to 74%. Merlo concluded that a cut-off value of 500IU/ml was an ideal threshold for predicting PDS success (59), which was consistent with the result of previous research. The study by Rodriguez et al. showed that patients with a pre-treatment CA-125 level of ≤100U/mL were more likely to undergo cytoreductive surgery without residual lesions (60). Lin et al, on the other hand, proposed that a pretreatment serum CA-125 level of ≥35 U/mL is an independent prognostic factor in epithelial ovarian cancer and that elevated levels should be regarded as a high risk of recurrence and mortality (25).

Based on our findings and those of other relevant studies, pre-treatment CA-125 may contribute to the prognosis of cancer and the development of clinical treatment strategies. For example, EOC patients with low pre-treatment CA-125 may benefit more from PDS, NACT, or other cancer-related treatments than EOC patients with high CA-125. The prognostic relevance of pre-treatment CA-125 has attracted clinical attention, and in order to benefit EOC patients, ongoing and systematic studies are still necessary to fully understand and utilize CA-125.

There are several limitations to this study. First, the number of studies we included was relatively small. Second, we were unable to perform further subgroup analyses due to the diversity of cut-off values defined in each study. Third, most of the enrolled studies were retrospective and extracted several HRs from univariate analyses, which may lead to overestimated effect sizes. Therefore, this study may have information bias, selection bias, and misclassification bias, and the optimal cut-off value cannot be determined. Larger-scale prospective studies are needed to confirm the significance of pretreatment CA-125 in predicting the prognosis of EOC and to provide evidence for its clinical application. In summary, according to the current study, the pre-treatment CA-125 level has some certain significance in predicting the prognosis of EOC. The results of this study may help to determine whether patients may benefit from surgery, chemotherapy, and/or neoadjuvant chemotherapy.

## Conclusion

This meta-analysis has shown that higher pre-treatment CA-125 levels are significantly associated with poorer OS and PFS in patients with EOC. We suggest that CA-125 should be monitored to guide prognosis and provide reliable information on the risk of disease progression in EOC. Nevertheless, due to some limitations of this study, large multi-center prospective trials are needed to examine the prognostic role of pretreatment CA-125 in EOC.

## Data Availability Statement

The original contributions presented in the study are included in the article/**Supplementary Material**. Further inquiries can be directed to the corresponding author.

## Author Contributions

QW designed this study. QW and SZ contributed to literature retrieval, review and data extraction. SZ and XL conducted the statistical analyses. QW, and XL contributed to the manuscript drafting. QW, and XL contributed to the manuscript revision. XF provided fund help. All authors contributed to the article and approved the submitted version.

## Funding

Project supported by the National Natural Science Foundation of China (Grant NO.81973894).

## Conflict of Interest

The authors declare that the research was conducted in the absence of any commercial or financial relationships that could be construed as a potential conflict of interest.

## Publisher’s Note

All claims expressed in this article are solely those of the authors and do not necessarily represent those of their affiliated organizations, or those of the publisher, the editors and the reviewers. Any product that may be evaluated in this article, or claim that may be made by its manufacturer, is not guaranteed or endorsed by the publisher.
