# Supplementary material for: Prognostic Value of Elevated Pre-treatment Serum CA-125 in Epithelial Ovarian Cancer: A Meta-Analysis
Source: Front Oncol. 2022 Apr 7;12:868061. doi: 10.3389/fonc.2022.868061 (PMC9022002; doi:10.3389/fonc.2022.868061)

**Table S1. English search strategies**

| PubMed | ((((((((((((((((((((Neoplasm, Ovarian[Title/Abstract]) OR (Ovar* Neoplasm*[Title/Abstract]))) OR (Neoplasm*, Ovary[Title/Abstract])))) OR (Neoplasms, Ovarian[Title/Abstract])) OR (Ovary Cancer*[Title/Abstract])) OR (Cancer, Ovary[Title/Abstract])) OR (Cancers, Ovary[Title/Abstract]))) OR (Ovarian Cancer[Title/Abstract])) OR (Cancer*, Ovarian[Title/Abstract]))) OR (Ovarian Cancers[Title/Abstract])) OR (Cancer of Ovary[Title/Abstract])) OR (Cancer of the Ovary[Title/Abstract])) OR (Ovary tumor[Title/Abstract])) OR ("Ovarian Neoplasms"[Mesh])) AND (((CA-125[Title/Abstract]) OR (CA 125[Title/Abstract])) OR (CA125[Title/Abstract]) OR ("Carbohydrate antigen 125"[Title/Abstract]) OR ("Cancer antigen 125"[Title/Abstract]))) AND ((Prognos*[Title/Abstract]) OR (Predict*[Title/Abstract])) |
| --- | --- |
| Web of Science | #6 #4  AND  #5  #5 ALL=neoadjuvant  #4 #1  AND  #2  AND  #3  #3 TS=(Prognos*)  OR  TS=(Predict*)  #2 TS=(CA-125)  OR  TS=(CA  125)  OR  TS=(CA125)  OR  TS=(Carbohydrate  antigen  125)  OR  TS=(Cancer  antigen  125)  #1 ((((((((((((((((((TS=(Ovarian Neoplasms)  )   OR   TS=(   Neoplasm,   Ovarian))   OR   TS=(Ovarian   Neoplasm))  OR   TS=(Ovary   Neoplasms))   OR   TS=(   Neoplasm,   Ovary))   OR  TS=(   Neoplasms,   Ovary))   OR   TS=(   Ovary   Neoplasm))   OR  TS=(   Neoplasms,   Ovarian))   OR   TS=(   Ovary   Cancer))   OR  TS=(   Cancer,   Ovary))   OR   TS=(   Cancers,   Ovary))   OR  TS=(   Ovary   Cancers))   OR   TS=(Ovarian   Cancer))   OR  TS=(Cancer,   Ovarian))   OR   TS=(   Cancers,   Ovarian))   OR  TS=(   Ovarian   Cancers))   OR   TS=(   Cancer   of   Ovary))   OR  TS=(   Cancer   of   the   Ovary))   OR   TS=(   Ovary   tumor) |
| Embase | ('ovary tumor'/exp OR 'neoplasm, ovarian':ab,ti OR 'ovarian neoplasm':ab,ti OR 'ovary neoplasms':ab,ti OR 'neoplasm, ovary':ab,ti OR 'neoplasms, ovary':ab,ti OR 'ovary neoplasm':ab,ti OR 'neoplasms, ovarian':ab,ti OR 'ovary cancer':ab,ti OR 'cancer, ovary':ab,ti OR 'cancers, ovary':ab,ti OR 'ovary cancers':ab,ti OR 'ovarian cancer':ab,ti OR 'cancer, ovarian':ab,ti OR 'cancers, ovarian':ab,ti OR 'ovarian cancers':ab,ti OR 'cancer of ovary':ab,ti OR 'cancer of the ovary':ab,ti OR 'ovarian neoplasms':ab,ti) AND ('ca 125':ab,ti OR ca125:ab,ti OR 'carbohydrate antigen 125':ab,ti OR 'cancer antigen 125':ab,ti) AND (predict*:ab,ti OR prognos*:ab,ti OR forecast*:ab,ti OR estimat*:ab,ti OR postdict*:ab,ti OR exist*:ab,ti OR surviv*:ab,ti OR 'prognosis'/exp OR 'prediction and forecasting'/exp) AND neoadjuvant |
| Cochrane | #1 MeSH descriptor: [Ovarian Neoplasms] explode all trees  #2 (Neoplasm, Ovarian):ti,ab,kw OR (Ovar* Neoplasm*):ti,ab,kw OR (Neoplasm*, Ovary):ti,ab,kw OR (Neoplasms, Ovarian):ti,ab,kw OR (Ovary Cancer*):ti,ab,kw  #3 (Cancer, Ovary):ti,ab,kw OR (Cancers, Ovary):ti,ab,kw OR (Ovarian Cancer):ti,ab,kw OR (Cancer*, Ovarian):ti,ab,kw OR (Ovarian Cancers):ti,ab,kw  #4 (Cancer of Ovary):ti,ab,kw OR (Cancer of the Ovary):ti,ab,kw OR (Ovary tumor):ti,ab,kw  #5 #1 or #2 or #3 or #4  #6 MeSH descriptor: [Prognosis] explode all trees  #7 (Prognos*):ti,ab,kw OR (Predict*):ti,ab,kw  #8 #6 or #7  #9 (CA-125):ti,ab,kw OR (CA125):ti,ab,kw OR (CA 125):ti,ab,kw OR (Carbohydrate antigen 125):ti,ab,kw OR (Cancer antigen 125):ti,ab,kw  #10 #5 and #8 and #9 |

Table S2 Subgroup analysis of pooled HRs and 95% CIs between pre-treatment serum CA-125 values and OS in EOC.

| **Variables** | **No. of studies** | **No. of patients** | **Effects model** | **HR (95% CI)** | **p** | **Heterogeneity I^2^, % p** | |
| --- | --- | --- | --- | --- | --- | --- | --- |
| **OS** |  |  |  |  |  |  | |
| **Total** | 23 | 6063 | random | 1.62(1.270-2.060) | ＜0.001 | 86.40% ＜0.001 | |
| **surgery+chemotherapy** | 11 | 5292 | random | 1.401(1.034-1.897) | ＜0.001 | 90.50% ＜0.001 |  |
| **surgery+chemotherapy+NACT** | 6 | 771 | random | 1.798(1.323-2.443) | 0.029 | 31.80% 0.197 |  |
| **I/II** | 2 | 636 | random | 2.238(1.400-3.577) | ＜0.001 | 0.00% 0.377 |  |
| **III/IV** | 5 | 3844 | random | 1.550(1.196-2.010) | 0.001 | 0.00% 0.972 |  |
| **I-IV** | 9 | 1512 | random | 1.725(1.366-2.179) | ＜0.001 | 37.90% 0.166 |  |

Supplementary Figure 1 | Sensitivity analysis of the effect of CA-125 on EOC (A) OS and (B) PFS.

(A)


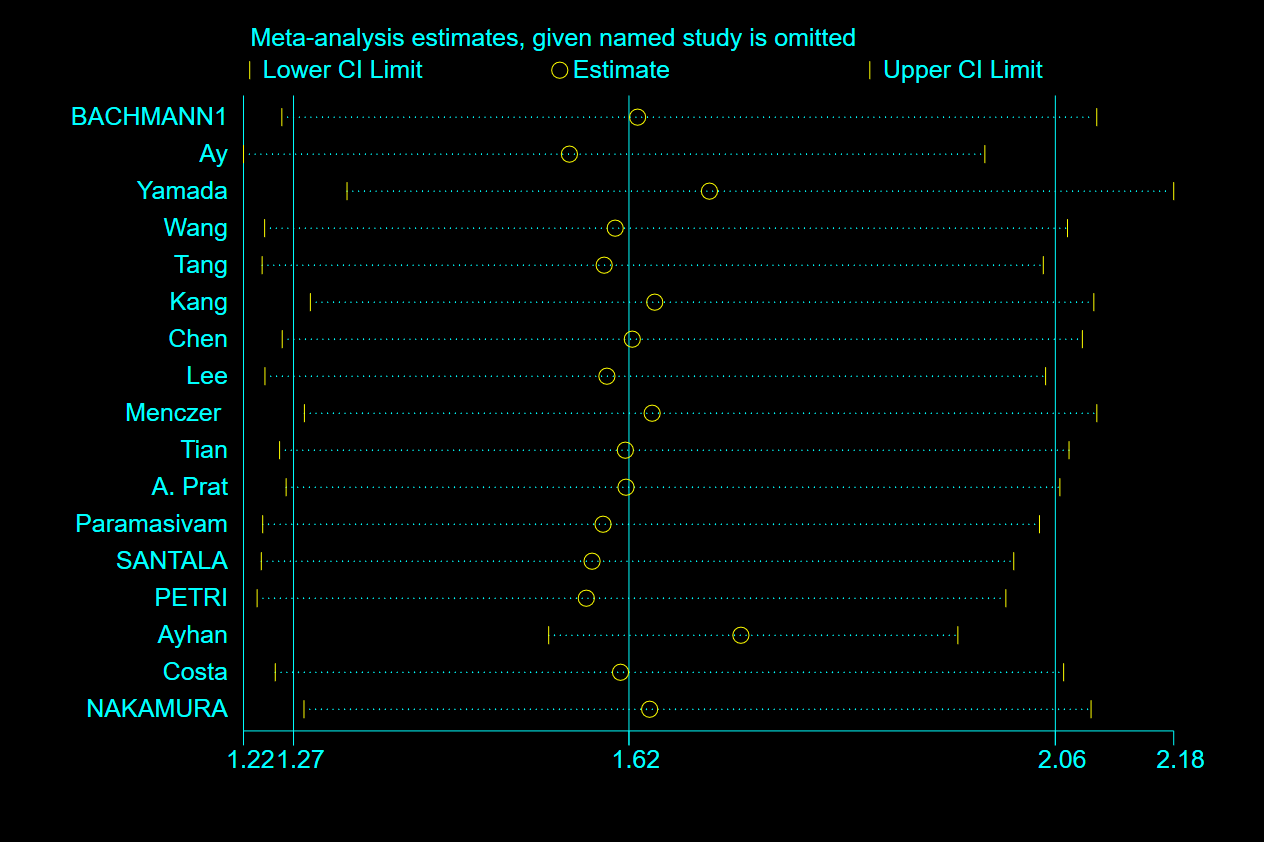


(B)


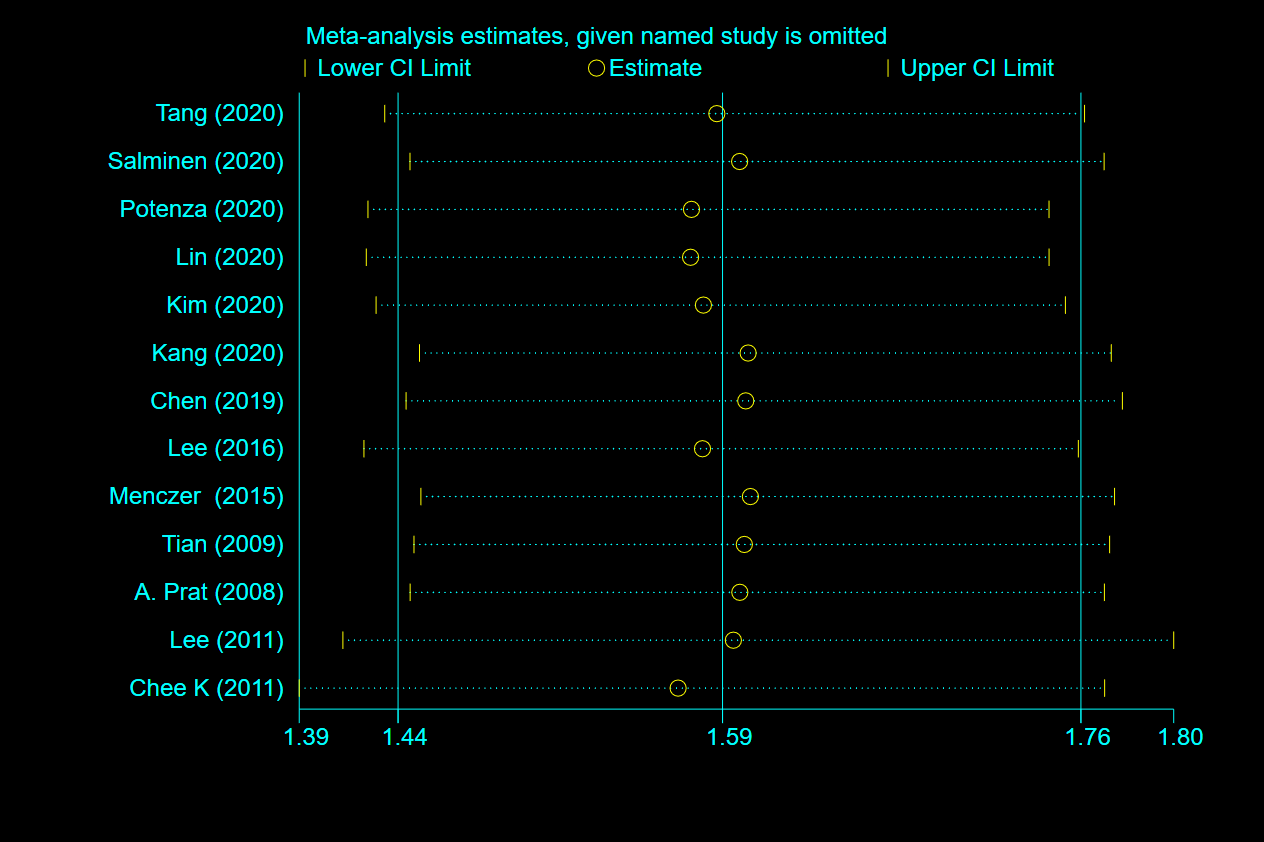


Supplementary Figure 2 | Publication bias tested by Begg’s test and Egger’s test. (A) Begg’s test for overall survival, p = 0.127. (B) Egger’s test for overall survival, p = 0.032.

(A)


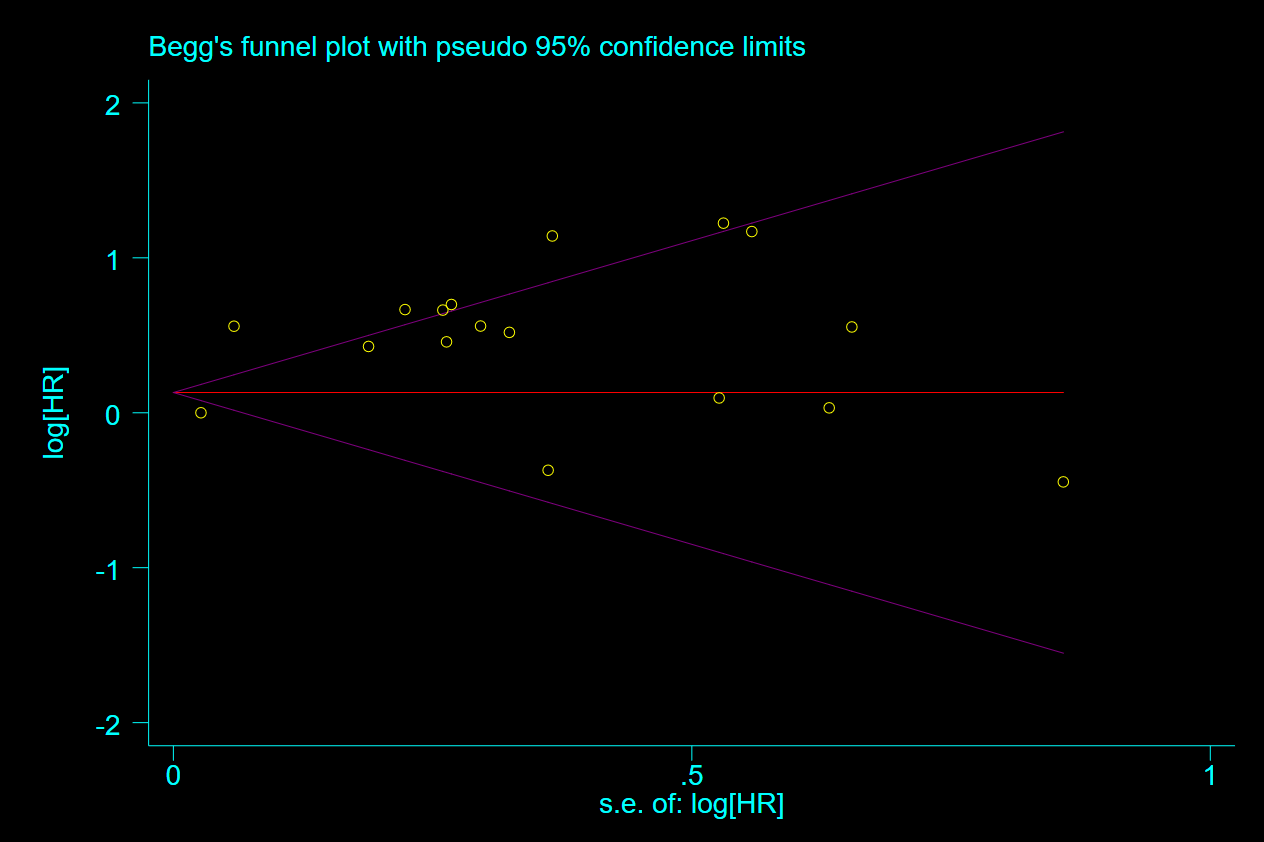


(B)


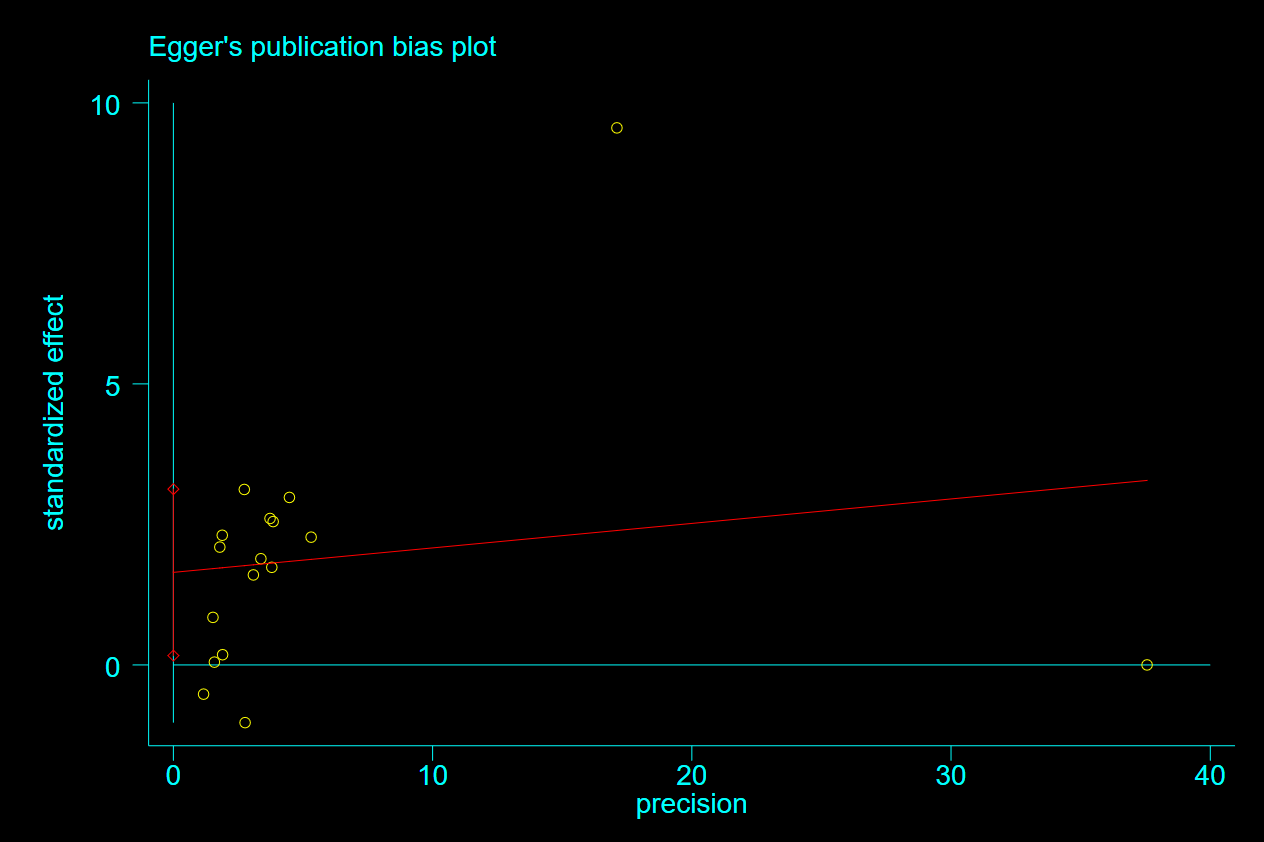


Supplementary Figure 3 | Funnel plot after trimming.


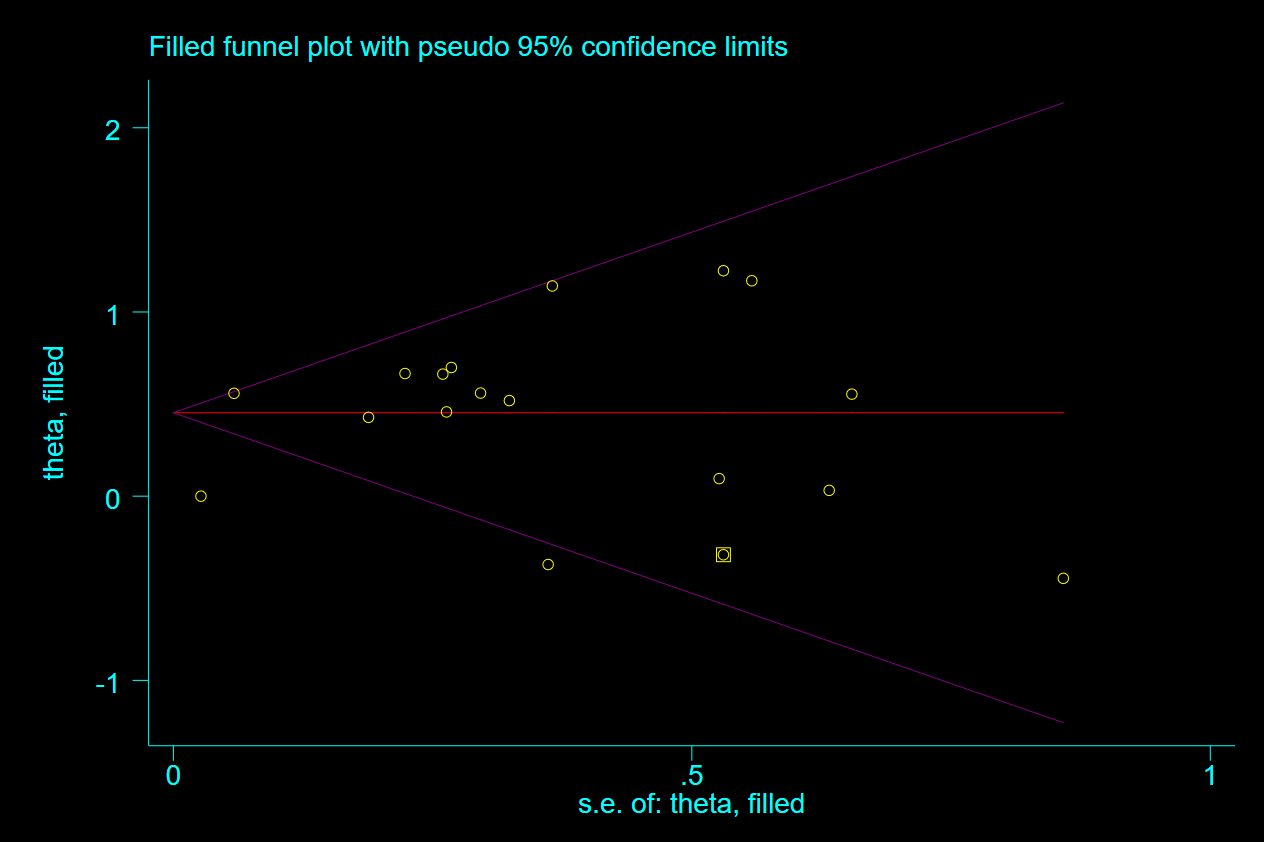

Supplement: Supplementary file 1 [file DataSheet_1.docx]
